# Whole Mitochondrial and Plastid Genome SNP Analysis of Nine Date Palm Cultivars Reveals Plastid Heteroplasmy and Close Phylogenetic Relationships among Cultivars

**DOI:** 10.1371/journal.pone.0094158

**Published:** 2014-04-09

**Authors:** Jamal S. M. Sabir, Dhivya Arasappan, Ahmed Bahieldin, Salah Abo-Aba, Sameera Bafeel, Talal A. Zari, Sherif Edris, Ahmed M. Shokry, Nour O. Gadalla, Ahmed M. Ramadan, Ahmed Atef, Magdy A. Al-Kordy, Fotoh M. El-Domyati, Robert K. Jansen

**Affiliations:** 1 Department of Biological Sciences, King Abdulaziz University, Jeddah, Saudi Arabia; 2 Department of Integrative Biology, University of Texas at Austin, Austin, Texas, United States of America; 3 Department of Genetics, Ain Shams University, Cairo, Egypt; 4 Department of Microbial Genetics, National Research Centre, Giza, Egypt; 5 Agriculture Research Center, Agricultural Genetic Engineering Research Institute, Giza, Egypt; 6 Department of Genetics and Cytology, National Research Centre, Dokki, Egypt; University of Hong Kong, China

## Abstract

Date palm is a very important crop in western Asia and northern Africa, and it is the oldest domesticated fruit tree with archaeological records dating back 5000 years. The huge economic value of this crop has generated considerable interest in breeding programs to enhance production of dates. One of the major limitations of these efforts is the uncertainty regarding the number of date palm cultivars, which are currently based on fruit shape, size, color, and taste. Whole mitochondrial and plastid genome sequences were utilized to examine single nucleotide polymorphisms (SNPs) of date palms to evaluate the efficacy of this approach for molecular characterization of cultivars. Mitochondrial and plastid genomes of nine Saudi Arabian cultivars were sequenced. For each species about 60 million 100 bp paired-end reads were generated from total genomic DNA using the Illumina HiSeq 2000 platform. For each cultivar, sequences were aligned separately to the published date palm plastid and mitochondrial reference genomes, and SNPs were identified. The results identified cultivar-specific SNPs for eight of the nine cultivars. Two previous SNP analyses of mitochondrial and plastid genomes identified substantial intra-cultivar ( = intra-varietal) polymorphisms in organellar genomes but these studies did not properly take into account the fact that nearly half of the plastid genome has been integrated into the mitochondrial genome. Filtering all sequencing reads that mapped to both organellar genomes nearly eliminated mitochondrial heteroplasmy but all plastid SNPs remained heteroplasmic. This investigation provides valuable insights into how to deal with interorganellar DNA transfer in performing SNP analyses from total genomic DNA. The results confirm recent suggestions that plastid heteroplasmy is much more common than previously thought. Finally, low levels of sequence variation in plastid and mitochondrial genomes argue for using nuclear SNPs for molecular characterization of date palm cultivars.

## Introduction

Date palm (*Phoenix dactylifera* L., Arecaceae) is the primary crop in many countries in western Asia and northern Africa [1]. This species is the oldest domesticated fruit-bearing tree with archaeological records dating back to 4000–5000 years ago in southern Iraq [2]–[3]. The cultivation of date palm enabled the development of the oasis system that allowed human expansion into the deserts of Asia and northern Africa [1]. The economic importance of date palm is due largely to its nutritionally valuable fruit, which contains 44–80% carbohydrates, 0.2–0.5% fat, 2.3–5.6% protein and 6–12% dietary fiber [4]–[5]. Numerous medicinal uses have also been attributed to date palms, including treatment for intestinal ailments, colds, sore throat, toothaches, fever, gonorrhea, and cough [6]–[8].

In view of the huge economic value of date palm, it is no surprise that there has been intense interest in breeding programs to enhance fruit production. However, there are several impediments to using traditional breeding practices for genetic improvement of new cultivars. Date palms are propagated either from seed or vegetative offshoots. For both approaches, extremely slow growth of seedlings and offshoots does not allow the use of classical breeding techniques; it takes 8–10 years before plants produce fruit. Propagation with seeds is unsuitable for commercial production because half of the progeny are males and there is currently no way to sex date palm plants at an early stage of development. The exact number of named date palm cultivars is uncertain but estimates as low as 340 and as high as 5000 have been reported [5], [9]. In the past, female cultivars have been identified by morphology of the fruits, including size, color, shape, and taste. Many of the named cultivars have local names that are based on geographic location resulting in names that may not be genetically distinct. During the past decade, there have been numerous attempts to use molecular markers to characterize date palm biodiversity but most of these studies have relied on fragment data, such as RAPD, ISSR, SSR, and AFLP approaches, e.g., [10]–[18]. Although these methods have some merit, they are not as reliable in characterizing genetic diversity and identifying cultivars as more recent genomic approaches [19]. The advent of next generation sequencing has generated a surge of interest in using genomic approaches to characterize date palm cultivars. The publication of the mitochondrial [20], nuclear [21]–[22], and plastid [23]–[24] genome sequences of date palm provides reference genomes to examine SNPs for identifying cultivar diversity and genetic relationships among cultivars.

Three recent studies utilized a whole genome approach to detect SNPs in the mitochondrial and plastid genomes of one or three common date palm cultivars [20], [23]–[24]. All of these studies were limited by the number of cultivars examined (3 or less) and issues concerning how to deal with the high percentage of the plastid genome that is also present as insertions in the mitochondrial genome (46.5%). All three studies concluded that there was considerable number of polymorphic sites in both the mitochondrial and plastid genes among and within the three cultivars examined. Two of these three studies [23]–[24] indicated that single plants were used for DNA isolation. In these cases, if intra-cultivar ( = intra-varietal) polymorphisms were present this would be unusual for plastid genomes because heteroplasmy has been considered to be rare [25], although more recent studies have suggested that it may be more common [26]–[27].

In this study, we sequenced mitochondrial and plastid genomes for nine additional date palm cultivars from Saudi Arabia. The four questions of our investigation are: (1) Is there heteroplasmy in the mitochondrial and plastid genomes?; (2) What is the effect of plastid DNA transfer to the mitochondrion on organellar SNP analyses? (3) Are organellar SNPs useful for identification of date palm cultivars?; and (4) What are the phylogenetic relationships among cultivars?

## Materials and Methods

### Sampling and DNA Isolation

Approximately 500 mg of field-collected leaf tissue from a single plant of each of the nine cultivars (Table 1) of *Phoenix dactylifera* was collected from Hada El-Sham Station, King Abdulaziz University, Saudi Arabia and frozen in liquid nitrogen. Isolation of total genomic DNA was performed using the modified procedure of Gawel and Jarret [28]. RNA contaminants were removed by adding 10 mg/ml of RNase A (Sigma, USA) to the DNA samples followed by incubation at 37°C for 30 min. Estimation of the DNA concentration was performed by measuring optical density at 260 nm according to the equation: DNA concentration (ug/ml) = OD260×50× dilution factor. Purified DNA samples were sent to Beijing Genomics Institute (BGI), Shenzhen, China for sequencing.

**Table 1:** Date palm cultivars examined.

Cultivar	Geographic location	Abbreviation	Sex	Fruit shape^1^	Fruit color^1^	Accession number
Sukkariat Al-Madinah	Al-Madinah	SUK-A	Female	Oval	Brown	SRR974792
Dekhaini Al-Riyadh	Al-Riyadh	DEK	Female	Cylindrical	Yellow	SRR974793
Ajwa Al-Madinah	Al-Madinah	AJW	Female	Oval	Red	SRR974754
Perny Al-Riyadh	Al-Riyadh	PER	Female	Oval	Brown	SRR974758
Sukkariat Qassim	Qassim	SUK-Q	male	Oval	Brown	SRR974794
Rabia Al-Madinah	Al-Madinah	RAB	male	Oval	Brown	SRR974795
Shalaby Al-Madinah	Al-Madinah	SHA	male	Cylindrical	Yellow	SRR974796
Moshwaq Al-Riyadh	Al-Riyadh	MOS-A	male	Cylindrical	Yellow	SRR974797
Moshwaq Hada Al-Sham	Hada Al-Sham	MOS-H	male	Cylindrical	Yellow	SRR974798
Khalas	Reference genome from GenBank	KHA-P; KHA-M	NI	NI	NI	NC_013991.2 – plastid; NC_016740.1 - mito

NI  =  Not included for reference genome; ^1^Fruit shape and color for male plants is based on these features from female plants of the same cultivar.

### Genome sequencing, mapping of reads to reference, and SNP analysis

Total genomic DNA was sequenced using the Illumina HiSeq 2000 platform at BGI. For each species, about 60 million 100 bp paired-end reads were generated from a sequencing library with 500 bp inserts. The raw data was processed in two steps: adapter sequences in reads were trimmed and then reads that contained more than 50% low quality bases (quality value ≤ 5) were removed. The remaining sequencing reads from the nine samples were aligned separately to the date palm plastid (NC_013991) and mitochondrial (NC_016740.1) reference genomes using BWA (http://bio-bwa.sourceforge.net/). Reads were then run through samtools version mpileup (http://samtools.sourceforge.net/) and bcftools pipelines to identify SNPs that are unique to the mitochondria or plastid genomes. Only SNPs with a read depth of ≥ 10, mapping quality≥20, and SNP quality≥15 were retained. Initially, all reads were included in the mapping but a separate mapping was performed after filtering out of all reads that aligned to both plastid and mitochondrial genomes; only filtered reads were used in all subsequent SNP comparisons.

### Alignment and phylogenetic analyses

Ten mitochondrial and plastid genomes (nine from this paper and one from GenBank, Table 1) were aligned with MAFFT [29]. These alignments were used to generate Maximum Likelihood trees using the PhyML plugin in Geneious 6.0.5 (Biomatters Ltd.). Congruence between trees generated from mitochondrial and plastid SNPs was examined using the incongruence length difference test (ILD) implemented in PAUP*4.0b10 [30].

## Results

### Mapping of reads to reference genomes

The number of reads generated for each sample ranged from 66.72 to 77.87 million. Mapping of the reads to the mitochondrial genome (NC_016740.1) covered 100% of the genome for all nine cultivars. The number of reads mapped to the mitochondrial genome varied from 854,270–1,495,892 depending on the cultivar, which included 1.41–2.07% of the total reads (Table 2). Mapping of the reads to the reference plastid genome (NC_013991.2) resulted in 99.61–100% coverage of the genome. The number of reads mapped to the plastid genome for the nine cultivars ranged from 751,281–1,153,632, which represents 0.96–1.52% of the total reads (Table 3).

**Table 2:** Summary of alignment results to the mitochondrial reference genome (NC_016740.1).

Cultivar	Total reads (million)	Number of reads mapped	Number of reads mapped after filtering	Coverage	Filtered coverage	% reads mapped	% reads mapped after filtering
SUK-A	72.23	1,495,892	985,521	418	276	2.07%	1.36%
DEK	77.87	1,096,970	712,383	307	199	1.41%	0.91%
AJW	75.38	1,484,793	972,198	415	272	1.97%	1.29%
PER	74.24	1,112,452	683,681	311	191	1.50%	0.92%
SUK-Q	66.72	854,270	454,897	239	127	1.28%	0.68%
RAB	75.8	1,167,048	627,419	326	176	1.54%	0.83%
SHA	72.35	1,049,708	645,157	294	180	1.45%	0.89%
MOS-A	70.3	1,016,158	637,760	284	178	1.45%	0.91%
MOS-H	74.95	1,127,157	692,700	315	194	1.50%	0.92%

**Table 3:** Summary of alignment results to the plastid reference genome (NC_013991.2).

Cultivar Name	Total reads (million)	Number of reads mapped	Number of reads mapped after filtering	Coverage	Filtered coverage	% reads mapped	% reads mapped after filtering
SUK-A	72.23	1,014,305	503,934	1,280	636	1.40%	0.70%
DEK	77.87	751,281	366,694	948	463	0.96%	0.47%
AJW	75.38	1,040,624	528,029	1,313	666	1.38%	0.70%
PER	74.24	853,777	425,006	1,078	536	1.15%	0.57%
SUK-Q	66.72	847,495	448,122	1,070	566	1.27%	0.67%
RAB	75.8	1,153,632	614,003	1,456	775	1.52%	0.81%
SHA	72.35	841,371	436,820	1,062	551	1.16%	0.60%
MOS-A	70.3	792,442	414,044	1,000	523	1.13%	0.59%
MOS-H	74.95	892,144	457,687	1,126	578	1.19%	0.61%

Since 10.3% of the 715,001 bp mitochondrial genome represents plastid insertions [20], the reads that mapped to both genomes were removed and the remaining reads were mapped to the reference plastid and mitochondrial genomes to avoid generating false SNPs that represent DNA sequences that were transferred from plastid genome to the mitochondrial genome. For the mitochondrial genome, this reduced the number of mapped reads to 627,419–985,521 for the nine cultivars, which represented 53–66% of the reads mapped before filtering (Table 2). In the case of the plastid genome, the number of reads mapped was reduced to 366,694–614,003 or 49–53% of the reads mapped before filtering (Table 3). Filtering out reads that mapped to both genomes reduced the number of SNPs detected in both the mitochondrial and plastid genomes and it also reduced the read depth coverage for each SNP.

### Mitochondrial SNPs

**Figure 1:**
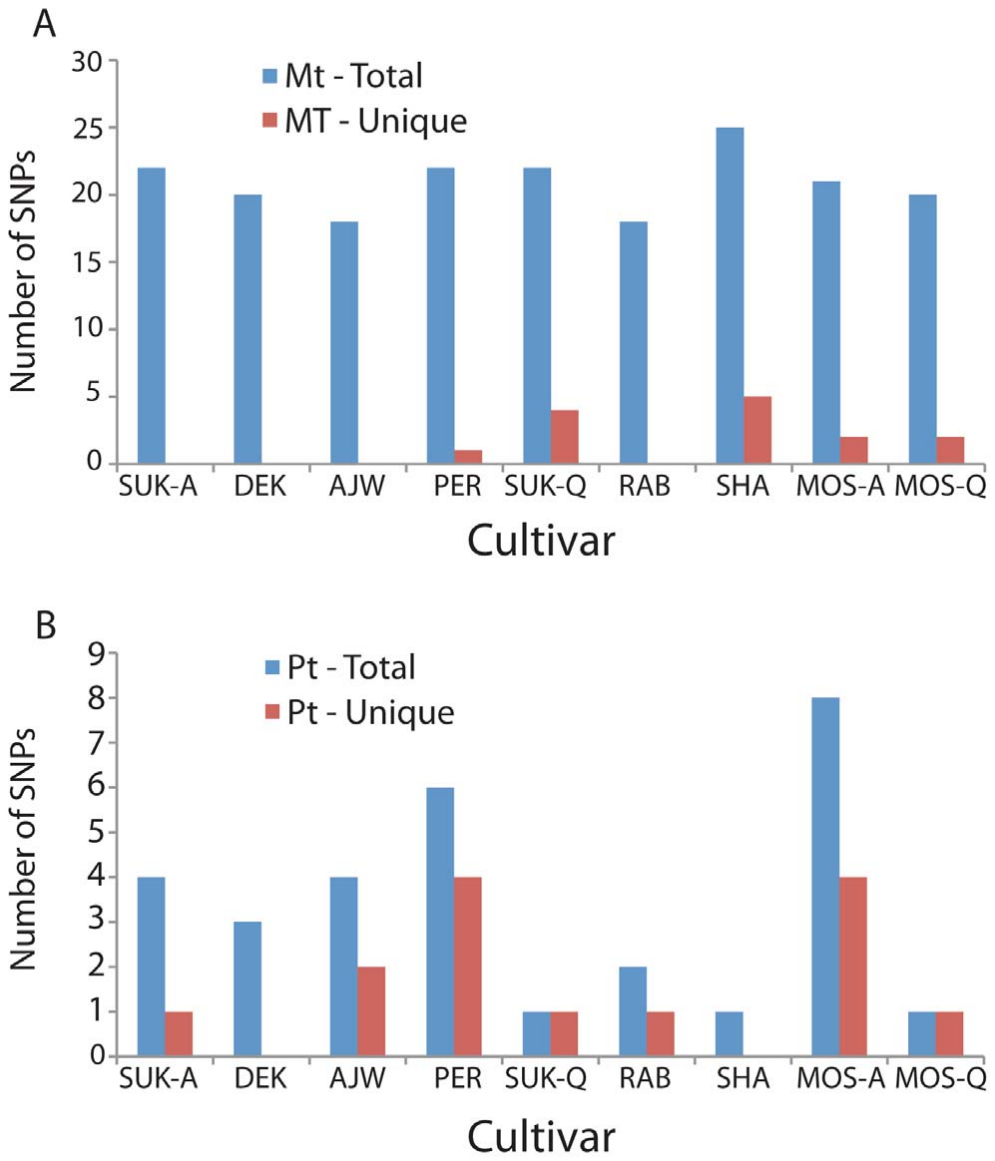
Number of total and unique SNPs detected for each of the nine Saudi Arabian date palm cultivars. (A) mitochondrial, (B) plastid.

**Figure 2:**
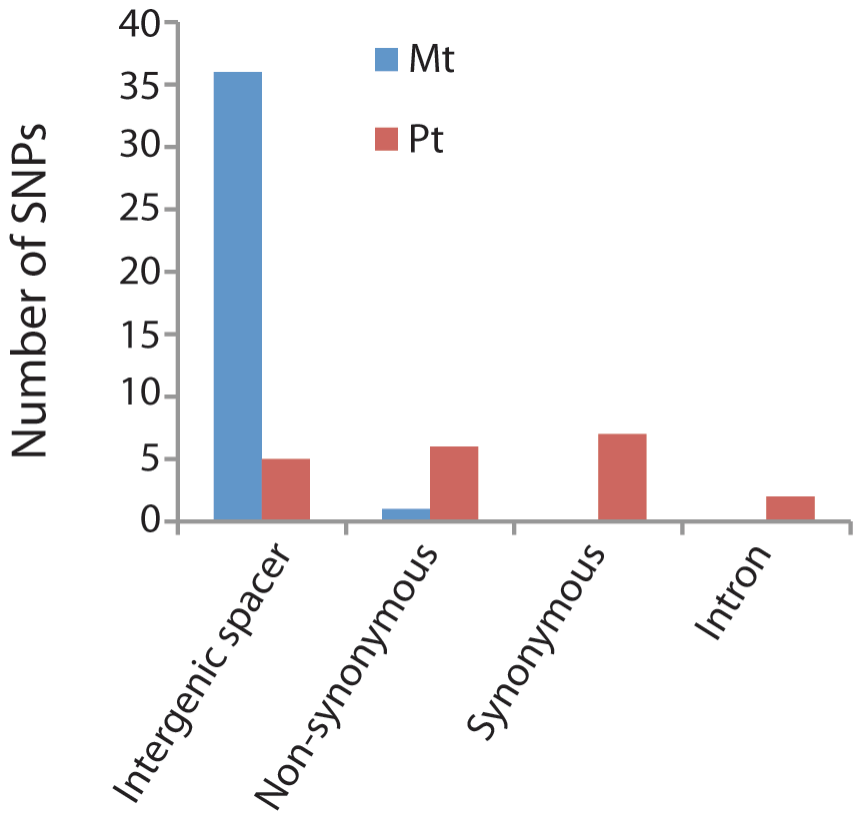
Number of mitochondrial and plastid SNPs in intergenic spacers, introns and protein coding genes. For those SNPs in coding regions the number that results in synonymous versus non-synonymous substitutions is indicated.

The number of mitochondrial SNPs detected for each of the nine Saudi Arabian date palm cultivars relative to the reference genome ranged from 18–25 for a total of 188 SNPs (Figure 1A). For the most part, mitochondrial SNPs were homogeneous since all reads at each SNP position had either the reference or the alternate nucleotide (Table 4). There were 15 SNPs that showed polymorphisms but in these cases the majority of the reads matched either the reference or the alternate nucleotide (Table 4). The 188 SNPs were located at 37 different sites in the mitochondrial genome. Most SNPs were shared among cultivars with only 14 unique to a single cultivar (Tables 4). The number of shared SNPs was 16 for all nine cultivars, two for eight cultivars, one for five cultivars, one for three cultivars, and three for two cultivars. Only five of the nine cultivars had unique mitochondrial SNPs that could be used as a marker for their identification (Figure 1A). All but one of the SNPs was located in intergenic spacer regions (Figure 2). The one exception was a nonsynonymous substitution in the *matR* gene at coordinate 559,552 in the cultivar Moshwaq Al-Riyadh (MOS-A).

**Table 4:** Mitochondrial SNPs sorted by position in the genome.

Cultivar	Position	Reference	Alternate	Quality	Read depth	Depth reference	Depth alternate	Location
SUK-A	117,620	G	A	78	21	0	20	IGS
DEK	117,620	G	A	61	13	0	11	IGS
AJW	117,620	G	A	69	14	0	13	IGS
PER	117,620	G	A	72	14	0	13	IGS
SUK-Q	117,620	G	A	65	10	0	10	IGS
RAB	117,620	G	A	81	16	0	14	IGS
SHA	117,620	G	A	81	21	0	21	IGS
MOS-A	117,620	G	A	73	12	0	10	IGS
MOS-H	117,620	G	A	62	13	0	11	IGS
SHA*	**130,703**	**C**	**T**	**34**	**20**	**9**	**7**	**IGS**
SHA*	**130,707**	**C**	**T**	**36**	**24**	**14**	**7**	**IGS**
SHA*	**130,715**	**G**	**A**	**95**	**32**	**22**	**8**	**IGS**
SUK-A	157,036	C	T	123	57	0	54	IGS
DEK	157,036	C	T	123	45	0	42	IGS
AJW	157,036	C	T	106	53	0	52	IGS
PER	157,036	C	T, G	100	37	0	35	IGS
SUK-Q	157,036	C	T	76	10	0	9	IGS
RAB	157,036	C	T	91	21	0	20	IGS
SHA	157,036	C	T	100	30	0	28	IGS
MOS-A	157,036	C	T	86	28	0	24	IGS
MOS-H	157,036	C	T	101	32	0	28	IGS
SUK-A	215,792	A	C	222	265	0	250	IGS
DEK	215,792	A	C	222	196	0	181	IGS
AJW	215,792	A	C	222	178	0	165	IGS
PER*	215,792	A	C	222	160	1	153	IGS
SUK-Q	215,792	A	C	206	84	0	75	IGS
RAB	215,792	A	C	218	123	0	113	IGS
SHA	215,792	A	C	222	118	0	109	IGS
MOS-A	215,792	A	C	222	132	0	127	IGS
MOS-H	215,792	A	C	222	142	0	131	IGS
SUK-Q*	**260,494**	**A**	**T**	**18**	**115**	**97**	**15**	**IGS**
MOS-H*	**329,782**	**G**	**A**	**60**	**21**	**16**	**5**	**IGS**
SUK-A	349,157	A	T	75	16	0	16	IGS
DEK	349,157	A	T	70	15	0	15	IGS
PER	349,157	A	T	73	15	0	14	IGS
**MOS-A**	**350,750**	**C**	**A**	**222**	**125**	**0**	**121**	**IGS**
MOS-H*	**452,157**	**C**	**G**	**38**	**159**	**129**	**22**	**IGS**
SUK-A	457,989	C	A	66	12	0	12	IGS
AJW	457,989	C	A	64	23	0	23	IGS
PER	457,989	C	A	79	18	0	16	IGS
SUK-Q	457,989	C	A	66	10	0	10	IGS
RAB	457,989	C	A	52	11	0	11	IGS
SHA	457,989	C	A	63	19	0	19	IGS
MOS-A	457,989	C	A	68	17	0	17	IGS
MOS-H	457,989	C	A	75	17	0	17	IGS
SUK-A	457,994	A	T	46	12	0	12	IGS
DEK	457,994	A	T	42	10	0	10	IGS
AJW	457,994	A	T	52	29	0	29	IGS
PER	457,994	A	T	63	20	0	19	IGS
SUK-Q	457,994	A	T	55	11	0	11	IGS
RAB	457,994	A	T	48	14	0	14	IGS
SHA	457,994	A	T	66	22	0	21	IGS
MOS-A	457,994	A	T	49	18	0	18	IGS
MOS-H	457,994	A	T	62	22	0	21	IGS
SUK-A	458,029	A	C	62	15	0	13	IGS
DEK	458,029	A	C	64	11	0	10	IGS
AJW	458,029	A	C	71	30	0	30	IGS
PER	458,029	A	C	63	20	0	20	IGS
SUK-Q	458,029	A	C	60	17	0	17	IGS
RAB	458,029	A	C	45	16	0	16	IGS
SHA	458,029	A	C	63	20	0	20	IGS
MOS-A	458,029	A	C	67	19	0	18	IGS
MOS-H	458,029	A	C	54	21	0	21	IGS
SUK-A	458,036	C	A	69	13	0	13	IGS
AJW	458,036	C	A	72	30	0	29	IGS
PER	458,036	C	A	79	20	0	18	IGS
SUK-Q	458,036	C	A	70	17	0	16	IGS
RAB	458,036	C	A	61	16	0	16	IGS
SHA	458,036	C	A	60	20	0	20	IGS
MOS-A	458,036	C	A	79	19	0	19	IGS
MOS-H	458,036	C	A	78	21	0	21	IGS
SUK-A	464,552	C	G	222	62	0	57	IGS
DEK	464,552	C	G	222	52	0	52	IGS
AJW	464,552	C	G	222	58	0	53	IGS
PER	464,552	C	G	222	57	0	51	IGS
SUK-Q	464,552	C	G	187	31	0	31	IGS
RAB	464,552	C	G	222	37	0	34	IGS
SHA	464,552	C	G	222	33	0	32	IGS
MOS-A	464,552	C	G	222	38	0	35	IGS
MOS-H	464,552	C	G	193	46	0	41	IGS
SUK-A	475,318	A	T	173	110	0	105	IGS
DEK	475,318	A	T	170	109	0	101	IGS
AJW	475,318	A	T	189	107	0	101	IGS
PER	475,318	A	T	163	93	0	88	IGS
SUK-Q	475,318	A	T	129	28	0	26	IGS
RAB	475,318	A	T	139	57	0	55	IGS
SHA	475,318	A	T	152	62	0	58	IGS
MOS-A	475,318	A	T	152	56	0	54	IGS
MOS-H	475,318	A	T	119	59	0	56	IGS
SUK-A	475,346	G	T	219	139	0	135	IGS
DEK	475,346	G	T	220	104	0	102	IGS
AJW	475,346	G	T	222	147	0	145	IGS
PER	475,346	G	T	207	98	0	97	IGS
SUK-Q	475,346	G	T	184	47	0	47	IGS
RAB	475,346	G	T	196	67	0	66	IGS
SHA	475,346	G	T	197	87	0	87	IGS
MOS-A	475,346	G	T	206	75	0	73	IGS
MOS-H	475,346	G	T	212	71	0	69	IGS
**SHA**	**482,322**	**A**	**C**	**93**	**11**	**0**	**11**	**IGS**
SUK-A	503,021	A	C	149	17	0	12	IGS
DEK	503,021	A	C	122	11	0	11	IGS
MOS-A*	**559,552**	**C**	**T**	**26**	**169**	**138**	**26**	**NS-matR**
SUK-Q*	**571,857**	**G**	**A**	**37**	**168**	**137**	**24**	**IGS**
SHA*	**572,726**	**G**	**A**	**22**	**194**	**152**	**31**	**IGS**
SUK-A	587,016	G	A	140	14	0	10	IGS
PER	587,016	G	A	102	10	0	6	IGS
SUK-Q*	**590,324**	**C**	**T**	**16**	**131**	**108**	**18**	**IGS**
SUK-Q*	**590,624**	**T**	**G**	**17**	**104**	**87**	**16**	**IGS**
PER*	**629,408**	**G**	**T**	**46**	**106**	**29**	**17**	**IGS**
SUK-A	632,571	A	C	88	105	0	96	IGS
DEK	632,571	A	C	91	91	0	75	IGS
AJW	632,571	A	C	76	89	0	79	IGS
PER	632,571	A	C	78	65	0	60	IGS
SUK-Q	632,571	A	C	68	45	0	39	IGS
RAB	632,571	A	C	85	64	0	56	IGS
SHA	632,571	A	C	67	51	0	48	IGS
MOS-A	632,571	A	C	62	47	0	39	IGS
MOS-H	632,571	A	C	75	70	0	64	IGS
SUK-A	642,650	G	T	99	50	0	45	IGS
DEK	642,650	G	T	149	47	0	36	IGS
AJW	642,650	G	T	146	43	0	30	IGS
PER	642,650	G	T	141	46	0	36	IGS
SUK-Q	642,650	G	T	104	20	0	14	IGS
RAB	642,650	G	T	136	24	0	21	IGS
SHA	642,650	G	T	119	39	0	31	IGS
MOS-A	642,650	G	T	128	41	0	34	IGS
MOS-H	642,650	G	T	124	40	0	27	IGS
SUK-A	642,669	T	G	210	51	0	50	IGS
DEK	642,669	T	G	222	49	0	48	IGS
AJW	642,669	T	G	222	43	0	42	IGS
PER	642,669	T	G	222	46	0	45	IGS
SUK-Q	642,669	T	G	222	20	0	20	IGS
RAB	642,669	T	G	222	26	0	24	IGS
SHA	642,669	T	G	222	41	0	37	IGS
MOS-A	642,669	T	G	222	42	0	41	IGS
MOS-H	642,669	T	G	222	41	0	40	IGS
SUK-A	642,689	A	T	107	50	0	49	IGS
DEK	642,689	A	T	137	46	0	45	IGS
AJW	642,689	A	T	130	42	0	41	IGS
PER	642,689	A	T	134	46	0	46	IGS
SUK-Q	642,689	A	T	141	19	0	19	IGS
RAB	642,689	A	T	137	28	0	26	IGS
SHA	642,689	A	T	141	43	0	40	IGS
MOS-A	642,689	A	T	157	43	0	42	IGS
MOS-H	642,689	A	T	153	41	0	41	IGS
DEK	642,706	G	T	75	39	0	9	IGS
SHA	642,706	G	T	63	34	0	7	IGS
SUK-A	642,707	A	C	27	39	0	5	IGS
DEK	642,707	A	C	58	39	0	8	IGS
PER	642,707	A	C	24.3	40	0	5	IGS
SHA	642,707	A	C	66	34	0	7	IGS
MOS-A	642,707	A	C	51	37	0	6	IGS
SUK-A*	658,617	T	G	222	294	1	263	IGS
DEK	658,617	T	G	218	206	0	188	IGS
AJW	658,617	T	G	222	326	0	290	IGS
PER	658,617	T	G	219	209	0	184	IGS
SUK-Q	658,617	T	G	195	131	0	120	IGS
RAB	658,617	T	G	220	187	0	168	IGS
SHA	658,617	T	G	222	206	0	185	IGS
MOS-A	658,617	T	G	216	180	0	165	IGS
MOS-H	658,617	T	G	222	202	0	187	IGS
SUK-A	711,571	T	G	105	108	0	98	IGS
DEK	711,571	T	G	98	81	0	72	IGS
AJW	711,571	T	G	103	94	0	86	IGS
PER*	711,571	T	G	87	72	1	63	IGS
SUK-Q	711,571	T	G	77	38	0	31	IGS
RAB	711,571	T	G	91	45	0	43	IGS
SHA	711,571	T	G, A	79	52	0	45	IGS
MOS-A	711,571	T	G	110	58	0	53	IGS
MOS-H	711,571	T	G	110	70	0	63	IGS
SUK-A	711,576	A	C	120	116	0	104	IGS
DEK	711,576	A	C	115	85	0	80	IGS
AJW	711,576	A	C	111	97	0	90	IGS
PER	711,576	A	C	104	74	0	66	IGS
SUK-Q	711,576	A	C	125	41	0	35	IGS
RAB	711,576	A	C	101	47	0	46	IGS
SHA	711,576	A	C	102	63	0	54	IGS
MOS-A	711,576	A	C	129	61	0	57	IGS
MOS-H	711,576	A	C	121	73	0	66	IGS
SUK-A	711,612	T	G	69	62	0	61	IGS
DEK	711,612	T	G	75	49	0	46	IGS
AJW	711,612	T	G	77	59	0	57	IGS
PER	711,612	T	G	72	46	0	44	IGS
SUK-Q	711,612	T	G	76	20	0	20	IGS
RAB	711,612	T	G	69	25	0	25	IGS
SHA	711,612	T	G	65	38	0	38	IGS
MOS-A	711,612	T	G	87	33	0	32	IGS
MOS-H	711,612	T	G	76	42	0	42	IGS

SNPs that are unique to an individual cultivar are in bold. * indicate polymorphic SNPs; IGS  =  intergenic spacer; NS  =  nonsynonymous.

### Plastid SNPs

The number of plastid SNPs ranged from one to eight per cultivar with a total of 30 among the nine date palm cultivars and all but two cultivars (DEK and SHA) had at least one unique SNP (Table 5; Figure 1B). One half of the SNPs (15) were present in a single cultivar with two shared by four cultivars, two by two cultivars, and one by three cultivars. All plastid SNPs were heterogeneous as evidenced by the fact that both the reference and alternate nucleotides were present in some of the reads (Table 5). For most SNPs the number of reads for each nucleotide were very similar indicating that date palm plastid genomes are heteroplasmic, especially since single plants were sampled for each cultivar. The 30 plastid SNPs were located in 20 different positions in the genome with 13 in genes, two in introns, and five in intergenic spacers (Table 5, Figure 2). For the genic SNPs six resulted in nonsynonymous changes and seven were synonymous substitutions (Figure 2).

**Table 5:** Plastid SNPs sorted by position in the genome.

Cultivar	Position	Reference	Alternate	Quality	Read Depth	Depth Reference	Depth Alternate	Location
SUK-A	12,167	A	C	21	96	9	7	S-atpA
DEK	12,191	A	C	58	134	9	16	S-atpA
MOS-A	12,191	A	C	22	140	13	8	S-atpA
PER	12,191	A	C	44	172	16	16	S-atpA
SUK-A	12,191	A	C	43	199	17	20	S-atpA
**MOS-A**	**38,157**	**T**	**G**	**52**	**11**	**4**	**5**	**S-psaB**
**MOS-A**	**38,160**	**C**	**T**	**36**	**11**	**5**	**5**	**S-psaB**
**MOS-A**	**38,181**	**A**	**C**	**56**	**13**	**6**	**7**	**S-psaB**
**PER**	**38,233**	**A**	**G**	**21**	**17**	**11**	**6**	**NS-psaB**
**PER**	**38,608**	**G**	**T**	**19.1**	**16**	**10**	**6**	**NS-psaB**
AJW	38,634	G	A	36	11	4	7	S-psaB
MOS-A	38,634	G	A	46	11	5	6	S-psaB
PER	38,634	G	A	70	21	11	10	S-psaB
SUK-A	38,634	G	A	55	14	7	7	S-psaB
**PER**	**38,692**	**T**	**G**	**49**	**11**	**4**	**5**	**NS-psaB**
DEK	40,739	G	A	33	27	12	10	NS-psaA
SHA	40,739	G	A	34	15	5	7	NS-psaA
**AJW**	**40,783**	**C**	**T**	**20**	**22**	**7**	**5**	**S-psaA**
AJW	40,785	C	T	33	22	9	5	NS-psaA
DEK	40,785	C	T	27	23	11	6	NS-psaA
RAB	40,785	C	T	19.1	20	11	6	NS-psaA
**MOS-H**	**40,812**	**C**	**T**	**16.1**	**15**	**4**	**5**	**NS-psaA**
**AJW**	**48,066**	**A**	**C**	**23**	**14**	**7**	**6**	**I-trnL-UAA**
MOS-A	65,042	A	G	49	11	4	7	IGS-petA:psbJ
SUK-A	65,042	A	G	15.1	12	4	5	IGS-petA:psbJ
**MOS-A**	**65,045**	**A**	**T**	**22**	**11**	**4**	**7**	**IGS-petA:psbJ**
**MOS-A**	**65,409**	**C**	**G**	**19.1**	**38**	**30**	**7**	**IGS-petA:psbJ**
**SUK-Q**	**65,427**	**C**	**T**	**30**	**33**	**25**	**7**	**IGS-petA:psbJ**
**PER**	**65,453**	**G**	**A**	**58**	**42**	**26**	**13**	**IGS-petA:psbJ**
**RAB**	**79,175**	**G**	**T**	**18.1**	**22**	**14**	**7**	**I-petD**

SNPs that are unique to an individual cultivar are in bold. IGS  =  intergenic spacer; I  =  intron; NS  =  nonsynonymous; S  =  synonymous.

### Relationships among cultivars

**Figure 3:**
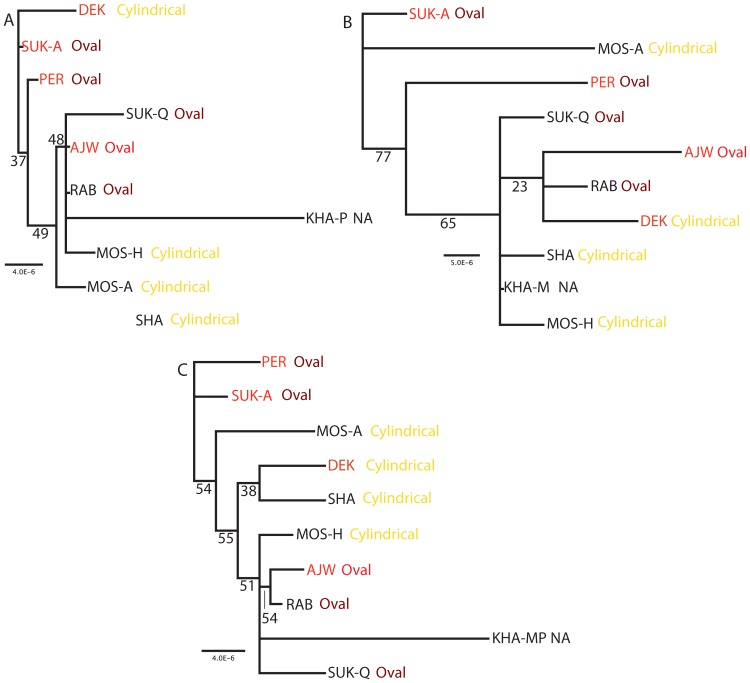
Maximum likelihood trees of mitochondrial SNPs for 10 date palm cultivars. (A), plastid (B), and combined (C). Numbers below each node represent bootstrap values for 1000 replicates. Cultivar abbreviations are provided in Table 1. Cultivar acronyms in red and black are female and male plants, respectively. Fruit shape is indicated and acronym names are color coded by fruit color (yellow, red, and brown).

Unrooted maximum likelihood (ML) trees were generated independently for mitochondrial and plastid SNPs to estimate relationships among the 10 cultivars of date palm (Figure 3). The mitochondrial tree (Figure 3A) was not well resolved and bootstrap support for resolved nodes was low. This is likely due to the fact that 30 of the 37 SNP positions were either unique to a single cultivar or shared by all nine cultivars relative to the reference (Table 4). Three features (sex, fruit shape, and fruit color) were plotted on the mitochondrial tree to determine if any of these characters corresponded to the relationships among cultivars (Figure 3A). The only feature that showed some correspondence with the tree topology was sex, with three of the four female plants examined grouping together.

The ML tree for the plastid SNPs was also not well-resolved or supported, however, bootstrap values for two nodes were slightly higher than those in the mitochondrial tree (Figure 3B). The low resolution and support was due to the small number of SNPs that are shared among a subset of the cultivars (Table 5). The topology of the plastid tree is largely incongruent with the mitochondrial tree and there is no correspondence between the tree topology and sex, fruit shape, or fruit color. However, ILD test for incongruence resulted in a p value = 0.63 indicating that the trees are not significantly incongruent. Therefore a combined analysis of mitochondrial and plastid SNPs was performed. The resulting ML tree topology was more resolved and better supported than either of the individual trees, however, there was still no correspondence between the ML tree topology and any of the three key features (Figure 3C).

## Discussion

### Heteroplasmy of SNPs

Three previous studies within and among one or three date palm cultivars reported intra- and inter-cultivar organellar SNPs [20], [23]–[24]. The detection of intra-cultivar SNPs suggests heteroplasmy in both mitochondrial and plastid genomes. Comparison of three date palm cultivars for mitochondrial SNPs using a combined Solid/454 sequencing approach from total genomic DNA revealed 347–378 intra-cultivar and 56–97 inter-cultivar SNPs [20]. However, the mitochondrial comparison did not account for the fact that 10.3% (73,691 bp or 46.5% of the plastid genome) of this genome represents DNA transferred from the plastid. Thus, it is likely that the high levels of intra-cultivar mitochondrial SNPs reported by Fang et al. [20] are due to the fact that sequences from the plastid genome mapped to the mitochondrial genome. In our SNP analysis of nine Saudi cultivars, filtering out all reads that mapped to both the mitochondrial and plastid genomes eliminated this artifact as evidenced by the fact that only 15 of the 188 mitochondrial SNPs remained polymorphic within individual cultivars (versus all of them before filtering), and in most of these cases the majority of the mapped reads matched either the reference or the alternate nucleotide (Table 4). Thus, intra-cultivar heteroplasmy in the mitochondrial genome of date palms is much less extensive than previously reported. We are not suggesting that intra-cultivar heteroplasmy does not exist, especially since it is recognized that heteroplasmy in plant mitochondria is common [31]–[32].

Straub et al. [33] raised concerns about reports of heteroplasmy in plastid genomes when performing next generation sequencing of total genomic DNA due to the transfer of plastid sequences to the nucleus and mitochondrion. Two previous studies examined SNPs in plastid genomes of date palms, one focused only on inter-cultivar variation [24] and the other on intra-cultivar variation [23]. Yang *et al.* [23] reported that all 78 SNPs are intra-cultivar, 16 in intergenic spacers and 62 in 23 different genes; in protein-coding genes 29 were synonymous substitutions and 31 were nonsynonymous. Yang *et al*. [23] utilized four adjustments in an attempt to eliminate false plastid SNPs caused by contamination of nuclear and mitochondrial sequences: (1) only count SNPs where the number of aligned reads is>50; (2) only count SNPs where the percentage of the reads with the minor variant is>10%; (3) exclude SNPs in regions where there are gaps in the alignment; and (4) eliminate SNPs in regions of overlapping homopolymer runs. The first adjustment will not take care of the problem of plastid DNA that has been transferred to the mitochondria because it is well known that read depth for plastid sequences is much higher due to the higher copy number of plastids [33]. So, one would predict that most SNPs in the mitochondrion that are in regions with inserted plastid DNA will have a much higher read depth because many plastid sequences would assemble to these mitochondrial regions. The fourth modification will only correct for errors associated with the well-characterized issue of homopolymer runs using the 454 sequencing platform. We took a much more conservative approach to testing for plastid heteroplasmy by eliminating all reads that mapped to both the plastid and mitochondrial genomes. Our setting of read depth for each SNP at≥10 would greatly reduce the chances of detecting mitochondrial sequences that have been transferred to the nucleus in the mitochondrial SNPs because the read depth of nuclear sequences is so much lower than either mitochondrial or plastid sequences [33]. Although this stringent constraint greatly reduced the number of SNPs detected and their read depth, all remaining plastid SNPs show heteroplasmy (Table 5). Furthermore, similar read depths for the reference and variant plastid SNPs (Table 5) support their location in the plastid genome as opposed to the nucleus, providing further support for occurrence of plastid heteroplasmy. Thus, it is clear that date palm plastid genomes are heteroplasmic, however, caution is recommended for SNP analyses using next generation sequencing of total genomic DNA.

The traditional view has been that heteroplasmy in plastids is uncommon [25] but several examples of this phenomenon have been detected across flowering plants, including in *Actinidia* [26], *Coreopsis* [34], *Cynomorium* [35], *Epilobium* [36], *Medicago* [37], [38], *Gossypium* [39], *Oenothera* [40] *Oryza* [41], *Passiflora* [42], *Pelargonium* [43], and *Senecio* [27]. Thus, heteroplasmy is more common than previously thought and it likely went undetected because of the paucity of molecular studies that examined intra-individual variation. Two different mechanisms have been suggested for the development of heteroplasmy in plastids. The more commonly suggested explanation is biparental inheritance in which each parent transmits organelles to the zygote, an inheritance mode that occurs in approximately one fifth of angiosperms [44]–[47]. The other mechanism occurs in plants with uniparental plastid inheritance in which plastid sorting in the parent is incomplete resulting in heteroplasmic gametes. In the case of date palm, incomplete sorting is the likely mechanism for heteroplasmy since plastid genomes are considered to have maternal inheritance [44]. We expect that many more cases of plastid heteroplasmy will be revealed as more genomic investigations of single plants are performed.

### Challenges of organellar SNP analysis caused by DNA transfers

Integration of plastid DNA into the mitochondrial genome can cause difficulties in utilizing organellar genomes for SNP analyses. In the previous studies of date palm organellar SNPs heteroplasmy was greatly overestimated because 10.3% of the mitochondrial genome represents plastid DNA transfers. The transfer of plastid DNA to the mitochondrion is a common phenomenon with 1–12% of published angiosperm mitochondrial genomes representing plastid DNA [48]. There are several approaches to dealing with this issue. Isolation of purified plastid or mitochondrial DNA would avoid this problem but it is often not possible to obtain sufficient plant material and/or isolate organellar DNA from many species. The most common approach for genomic SNP analyses is to sequence total genomic DNA and align these reads to a reference genome. Although it is well known that the depth of coverage for plastid reads is much higher than mitochondrial or nuclear reads, it is not likely that read depth could resolve this problem. Yang *et al*. [23] attempted this approach in the date palm investigation but they still overestimated the levels of intra-cultivar heterogeneity. We took a more stringent approach by removing all reads that mapped to both the mitochondrial and plastid genomes to attain a more realistic estimate of organellar SNPs among date palm cultivars. Although we are confident that we did not overestimate the number of intra-individual SNPs, the number of SNPs detected in both organellar genomes was greatly reduced due to the elimination of a large number of reads. In the case of date palm, nearly one half of the plastid genome (73,691 bp) has been transferred to the mitochondrial genome so the SNP analysis only sampled 53.5% of the plastid genome. Filtering reads that map to both organellar genomes is preferable to reporting erroneous SNPs caused by transfer of plastid DNA to the mitochondrion.

Another issue with using total genomic DNA for SNP analyses from genome sequence data is the prevalence of both plastid and mitochondrial DNA in the nucleus, which is commonly referred to as NUMTS (nuclear mtDNA) or NUPTs (nuclear ptDNA). In flowering plants, it is well known that large fragments of DNA from both of these genomes are transferred to the nucleus [49], and the proportion varies considerably among different species [50]. However, since the depth of reads for nuclear sequences is so much lower than for mitochondrial or plastid reads, read depth can be used to eliminate overestimation of the number of organellar SNPs.

### Cultivar identification and phylogenetic relationships

Date palm cultivar identification is complicated by the fact that there are so many named cultivars, and most of these are characterized by fruit size, color, shape, and taste. This has resulted in different cultivar names for the same morphological type in different countries. Also, reliance on characters that are only present on female plants has caused considerable confusion since it takes 8–10 years before plants flower. Thus, there has been an increasing effort to utilize molecular markers to define cultivars, and most of these studies have used fragment data from RAPD, ISSR, and AFLP comparisons. These approaches are problematic in terms of producing a well-characterized molecular signature for each cultivar, largely because of their limited repeatability. Even though the SNP comparison of the mitochondrial and plastid genomes was limited by cross compartment DNA transfer, our results were successful in detecting unique SNPs for eight of the nine cultivars examined (Figure 1). The main limitations of the organellar approach are the high levels of sequence conservation in these genomes and the need to eliminate regions of transferred plastid sequences to avoid erroneous SNP identification, which reduces the amount of sequence data available for cultivar identification. Two recent comparison of date palm SNPs in the nuclear genome provided much more data. Comparison of four cultivars by Al-Dous et al. [21] revealed over 3.5 million SNPs in 381 Mb and Al-Mssallem *et al.* [9] identified 3.85 to 6.63 SNPs per kb among 11 cultivars. Although transfer of mitochondrial and plastid DNA to the nucleus may complicate this approach, the huge number of SNPs in the nuclear genome makes this genome much more attractive for future characterization of date palm cultivars.

Phylogenetic analyses of mitochondrial and plastid SNPs generated incongruent tree topologies that provided only limited resolution among cultivars with low support values (Figure 3). This result is not surprising in view of the fact that a considerable portion of the data was filtered out of the analysis due to the transfer of 46.5% of the plastid genome to the mitochondrion. Expanded cultivar sampling is not likely to improve the situation. Only a few previous studies have utilized organellar genome sequences for SNP analyses within species [51]–[55], and in all cases the low level of variation detected limited the utility of this approach for population studies. In view of the much higher number of nuclear SNPs in date palms [9], [21], future phylogenetic analyses among cultivars should utilize this genome.

## References

[1] Jaradat AA (2011) Biodiversity of date palm, land use, land cover and soil sciences, In: Encyclopedia of Life Support Systems, Eolss Publishers, Oxford, UK, pp 1–31.

[2] Popenoe P (1973) The Date Palm. In: Field H (ed.) Field Research Projects, Coconut Grove, Miami, Florida.

[3] Zohary D, Spiegel-Roy P (1975) Beginnings of fruit growing in the Old World. Science 187: 319–327. 17814259 10.1126/science.187.4174.319

[4] Al-Shahib W, Marshall RJ (2003) The fruit of the date palm: its possible use as the best food for the future? Internat J Food Sci Nutr 54: 247–259. 10.1080/09637480120091982 12850886

[5] El Hadrami I, El Hadrami A (2009) Breeding date palm. In: Jain SM, Priyadarshan PM (eds.) Breeding Plantation Tree Crops, Springer, New York, pp 191–216.

[6] Hadrami El, Al-Kjhayri M (2012) Socioeconomic and traditional importance of date palm. Emirates J Food Agric 24: 371–385.

[7] Vayalil PK (2002) Antioxidant and antimutagenic properties of aqueous extract of date fruit (*Phoenix dactylifera* L. Arecaceae). J Agric Food Chem 50: 610–617. 11804538 10.1021/jf010716t

[8] Vayalil PK (2012) Date fruits (*Phoenix dactylifera* Linn.): An emerging medicinal food. Crit Rev Food Sci Nutr 52: 249–271. 22214443 10.1080/10408398.2010.499824

[9] Al-Mssallem IS (1996) Date Palm (*Phoenix dactylifera* L.) Vol. 7 , Encyclopedia Works Publishing & Distribution.

[10] Cao BR, Chao CT (2002) Identification of date palm cultivars in California using AFLP markers. HortScience 37: 966–968.

[11] Diaz S, Pire C, Ferrer J, Bonete MJ (2003) Identification of *Phoenix dactylifera* L. varieties based on amplified fragment length polymorphism (AFLP) markers. Cell Mol Biol Lett 8: 891–899. 14668912

[12] El-Khishin DA, Adawy SS, Hussein EHA, El-Itriby HA (2003) AFLP fingerprinting of some Egyptian date palm (*Phoenix dactylifera* L.) cultivars. Arab J Biotech 6: 223–234.

[13] Adawy SS, Hussein EHA, Ismail SME, El-Itriby HA (2005) Genomic diversity in date palm (*Phoenix dactylifera* L.) as revealed by AFLPs in comparison to RAPDs and ISSRs. Arab J Biotech 8: 99–114.

[14] Younis RAA, Ismail OM, Soliman SS (2008) Identification of sex-specific DNA markers for date palm (*Phoenix dactylifera* L.) using RAPD and ISSR techniques. Res J Agric Biol Sci 4: 278–284.

[15] Abdulla M, Gamal O (2010) Investigation on molecular phylogeny of some date palm (*Phoenix dactylifera* L.) cultivars by protein, RAPD and ISSR markers in Saudi Arabia. Austral J Crop Sci 4: 23–28.

[16] Moghaieb REA, Abdel- Hadi AA, Ahmed MRA, Hassan AGM (2010) Genetic diversity and sex determination in date palms (*Phoenix dactylifera* L.) based on DNA markers. Arab J Biotech 13: 143–156.

[17] Al-Mahmoud ME, Al-Dous EK, Al-Azwani EK, Malek JA (2012) DNA-based assays to distinguish date palm (Arecaceae) gender. Amer J Bot e7–e10. 10.3732/ajb.1100425 22203652

[18] Sabir J, Abo-Aba S, Bafeel S, Edris S, Shokry AM, et al (2014) Characterization of ten date palm (*Phoenix dactylifera* L.) cultivars from Saudi Arabia using AFLP and ISSR markers. C R Biologies 337: 6–18. 24439547 10.1016/j.crvi.2013.11.003

[19] Sharma R, Joshi A, Maloo SR, Rajaman G (2012) Assessment of genetic finger printing using molecular marker in plants: A Review. Sci Res Impact 1: 29–36.

[20] Fang Y, Wu H, Zhang T, Yang M, Yin Y, et al (2012) A complete sequence and transcriptomic analyses of Date palm (*Phoenix dactylifera* L.) mitochondrial genome. PLoS ONE 7: e37164. 22655034 10.1371/journal.pone.0037164 PMC3360038

[21] Al-Dous EK, George B, Al-Mahmoud ME, Al-Jaber, Wang H, et al (2011) De novo genome sequencing and comparative genomics of date palm (*Phoenix dactylifera*). Nature Biotech 29: 521–527. 10.1038/nbt.1860 21623354

[22] Al-Mssallem IS, Hu S, Zhang X, Lin Q, Liu W, et al. (2013) Genome sequence of the date palm *Phoenix dactylifera* L. Nature Commun doi:10.1038/ncomms3274. 10.1038/ncomms3274 PMC3741641 23917264

[23] Yang M, Zhang X, Liu G, Yin Y, Yun Q, et al (2010) The complete chloroplast genome sequence of date palm (*Phoenix dactylifera* L.). PLoS One 5: e12762. 20856810 10.1371/journal.pone.0012762 PMC2939885

[24] Khan A, Khan IA, Heinze B, Azim MK (2012) The chloroplast genome sequence of date palm (*Phoenix dactylifera* L. cv. ‘Aseel’). Plt Mol Biol Rep 30: 666–678.

[25] Birky CW (1983) Relaxed Cellular Controls and Organelle Heredity. Science 222: 468–475. 6353578 10.1126/science.6353578

[26] Chat J, Decroocq S, Decroocq V, Petit RJ (2002) A case of chloroplast heteroplasmy in Kiwifruit (*Actinidia deliciosa*) that is not transmitted during sexual reproduction. J Hered 93: 293–300. 12407220 10.1093/jhered/93.4.293

[27] Frey JE, Müller-Schärer H, Frey B, Frei D (1999) Complex relation between triazine-susceptible phenotype and genotype in the weed *Senecio vulgaris* may be caused by chloroplast DNA polymorphism. Theor Appl Genet 99: 578–586. 22665192 10.1007/s001220051271

[28] Gawel NJ, Jarret RL (1991) A modified CTAB DNA extraction procedure for *Musa* and *Ipomoea* . Plant Mol Biol Rep 9: 262–266.

[29] Katoh S (2011) MAFFT multiple sequence alignment software version 7: Improvements in performance and usability. Mol Biol Evol 30: 772–780. 10.1093/molbev/mst010 PMC3603318 23329690

[30] Swofford DL (2003) PAUP*, Phylogenetic Analysis Using Parsimony (* and other methods), ver. 4.0b10. Sinauer Associates, Sunderland MA.

[31] Kmiec B, Woloszynska M, Janska H (2006) Heteroplasmy as a common state of mitochondrial genetic information in plants and animals. Curr Genet 50: 149–159. 16763846 10.1007/s00294-006-0082-1

[32] Arrieta-Montiel MP, Mackenzie SA (2011) Plant mitochondrial genomes and recombination. In: Kempken F (ed.) Plant Mitochondria, Springer, New York, pp 65–84.

[33] Straub SCK, Parks M, Weitemier K, Fishbein M, Cronn RC, et al (2012) Navigating the tip of the genomic iceberg: next-generation sequencing for plant systematics. Amer J Bot 99: 349–364. 22174336 10.3732/ajb.1100335

[34] Mason RJ, Holsinger KE, Jansen RK (1994) Biparental inheritance of the chloroplast genome in *Coreopsis grandiflora* (Asteraceae). J Hered 85: 171–173.

[35] Garcia MA, Nicholson EH, Nickrent DL (2004) Extensive intraindividual variation in plastid rDNA sequences from the holoparasite *Cynomorium coccineum* (Cynomoriaceae). J Mol Evol 58: 322–332. 15045487 10.1007/s00239-003-2554-y

[36] Michaelis P (1962) Uber gehäufte Plastidenabänderungen I. Biol Zentralblatt 81: 91–128.

[37] Lee DJ, Blake TK, Smith SE (1988) Biparental inheritance of chloroplast DNA and the existence of heteroplasmic cells in alfalfa. Theor Appl Genet 76: 545–549. 24232273 10.1007/BF00260905

[38] Johnson LB, Palmer JD (1989) Heteroplasmy of chloroplast DNA in *Medicago* . Plt Mol Biol 12: 3–11. 10.1007/BF00017442 24272712

[39] Lax AR, Vaughn KC, Duke SO, Endrizzi JE (1987) Structural and physiological studies of a plastome cotton mutant with slow sorting out. J Hered 78: 147–152.

[40] Chiu W-L, Stubbe W, Sears BB (1988) Plastid inheritance in *Oenothera*: Organelle genome modifies the extent of biparental plastid transmission. Curr Genet 13: 181–189.

[41] Moon E, Kao TH, Wu R (1987) Rice chloroplast DNA molecules are heterogeneous as revealed by DNA sequences of a cluster of genes. Nucl Acids Res 15: 611–630. 3029686 10.1093/nar/15.2.611 PMC340455

[42] Hansen AK, Escobar LE, Gilbert LE, Jansen RK (2007) Paternal, maternal, and biparental inheritance of the chloroplast genome in *Passiflora* (Passifloraceae): Implications for phylogenetic studies. Amer J Bot 94: 42–46. 21642206 10.3732/ajb.94.1.42

[43] Tilney-Bassett RAE, Birky CW Jr (1981) The mechanism of the mixed inheritance of chloroplast genes in *Pelargonium*. Evidence from gene frequency distributions among the progeny of crosses. Theor Appl Genet 60: 43–53. 24276587 10.1007/BF00275177

[44] Corriveau JL, Coleman AW (1988) Rapid screening method to detect potential biparental inheritance of plastid DNA and results for over 200 angiosperms. Amer J Bot 75: 1443–1458.

[45] Mogensen HL (1996) The hows and whys of cytoplasmic inheritance in seed plants. Amer J Bot 83: 383–404.

[46] Zhang Q, Liu Y (2003) Sodmergen (2003) Examination of the cytoplasmic DNA in male reproductive cells to determine the potential for cytoplasmic inheritance in 295 angiosperm species. Plt Cell Physiol 44: 941–951. 10.1093/pcp/pcg121 14519776

[47] Hagemann R (2004) The sexual inheritance of plant organelles. In: Daniell HD, Chase C (eds) Molecular Biology and Biotechnology of Plant Organelles, Chloroplasts and Mitochondria, Springer, New York, pp 2–31.

[48] Mower JP, Sloan DB, Alverson AJ (2012) Plant mitochondrial genome diversity: the genomics revolution. In: Wendel JF, Greilhuber J, Dolezel J, Leitch IJ (eds) Plant Genome Diversity Volume 1: Plant Genomes, their Residents, and their Evolutionary Dynamics, Springer-Verlag, Wien, pp 123–144.

[49] Noutsos C, Richly E, Leister D (2005) Generation and evolutionary fate of insertions of organelle DNA in the nuclear genomes of flowering plants. Genome Res 15: 616–628. 15867426 10.1101/gr.3788705 PMC1088290

[50] Michalovova M, Vyskot B, Kejnovsky E (2013) Analysis of plastid and mitochondrial DNA insertions in the nucleus (NUPTs and NUMTs) of six plant species: size, relative age and chromosomal localization. Heredity 111: 314–320. 23715017 10.1038/hdy.2013.51 PMC3807264

[51] Tian X, Zheng J, Hu S, Yu J (2006) The rice mitochondrial genomes and their variations. Plt Physiol 140: 401–410. 10.1104/pp.105.070060 PMC1361312 16384910

[52] Whittall JB, Syring J, Parks M, Buenrostro J, Dick C, et al (2012) Finding a (pine) needle in a haystack: chloroplast genome sequence divergence in rare and widespread pines. Mol Ecol 19: 100–114. 10.1111/j.1365-294X.2009.04474.x 20331774

[53] Doorduin L, Gravendeel B, Lammers Y, Ariyurek Y, Chin-A-Woeng T, et al (2011) The complete chloroplast genome of 17 individuals of pest species *Jacobaea vulgaris*: SNPs, microsatellites and barcoding markers for population and phylogenetic studies. DNA Res 18: 93–105. 21444340 10.1093/dnares/dsr002 PMC3077038

[54] McPherson H, van der Mere M, Delaney SK, Edwards MA, Henry RJ, et al (2013) Capturing chloroplast variation for molecular ecology studies: a simple next generation sequencing approach applied to a rainforest tree. BMC Ecol 13: 8. 23497206 10.1186/1472-6785-13-8 PMC3605380

[55] Melodelima C, Lobréaus S (2013) Complete *Arabis alpina* chloroplast genome sequence and insight into its polymorphism. Meta Gene 1: 65–75. 25606376 10.1016/j.mgene.2013.10.004 PMC4205033

